# Barbed Sutures Are Associated With a Lower Incidence of Incisional Hernia Compared With Continuous Non‐Barbed Monofilament Sutures After Minimally Invasive Gastroenterological Surgery

**DOI:** 10.1002/ags3.70294

**Published:** 2026-10-01

**Authors:** Shusaku Honma, Takashi Takashima, Tatsuhi Ushikubo, Kana Ishikawa, Takashi Kumode, Takahisa Suzuki, Teppei Murakami, Shinichi Hosokawa, Sanae Nakajima

**Affiliations:** ^1^ Department of Surgery Kobe City Medical Center General Hospital Kobe Hyogo Japan; ^2^ Department of Surgery Kobe City Medical Center West Hospital Kobe Hyogo Japan

**Keywords:** barbed sutures, gastroenterological surgery, incisional hernia, minimally invasive surgery, rectus diastasis

## Abstract

**Aim:**

Although barbed sutures have been proposed to improve fascial closure, their superiority over conventional, continuous, slowly absorbable sutures remains unclear. Therefore, we compared the incidence of incisional hernia (IH), a common complication of minimally invasive gastroenterological surgery, after using the two suture types.

**Methods:**

We retrospectively analyzed patients who underwent elective minimally invasive gastroenterological surgery requiring a midline incision between 2021 and 2025. The patients were divided into barbed suture (ST) and continuous slowly absorbable monofilament suture (PC) groups. The primary outcome was the incidence of IH at 1 year, as assessed using computed tomography. Inverse probability of treatment weighting (IPTW) based on propensity scores was used to balance the baseline characteristics.

**Results:**

A total of 255 patients were included in this study (142 and 113 in the PC and ST groups, respectively). IPTW adjustment balanced the baseline characteristics. The incidence of IH at 1 year was significantly lower in the ST group than that in the PC group. Weighted logistic regression demonstrated that barbed sutures were associated with a reduced risk of IH (odds ratio, 0.46; 95% confidence interval, 0.22–0.99; *p* = 0.047). Among the patients who developed IH, the defect size did not differ between the groups, although all symptomatic cases occurred in the PC group.

**Conclusions:**

Barbed sutures are associated with a lower incidence of IH than conventional continuous sutures after minimally invasive gastroenterological surgery. Therefore, in addition to suture selection, appropriate closure techniques and risk stratification may be important in preventing IH.

## Graphical abstract

Incisional hernia remains a common complication after minimally invasive gastroenterological surgery despite small extraction incisions. In this propensity score‐weighted analysis, barbed sutures significantly reduced the incidence of incisional hernia compared with conventional slowly absorbable monofilament sutures without increasing postoperative complications. Barbed sutures may therefore provide an effective strategy for preventing incisional hernia following minimally invasive gastroenterological surgery.

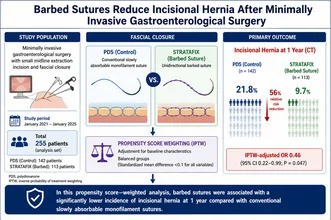

## 1 Introduction

Incisional hernia (IH), one of the most frequent postoperative complications, results in pain and cosmetic concerns and carries the risk of small bowel incarceration, which requires emergency surgical intervention. Even in the absence of incarceration, surgical repair is often necessary for definitive treatment, which imposes additional physical and economic burdens on patients [1]. Recent advances in surgical techniques have enabled abdominal surgeries to be performed using minimally invasive approaches, such as conventional multiport, robot‐assisted, or single‐incision laparoscopic surgery. Compared to open surgery, laparoscopic approaches are associated with a reduced risk of IH [2]. However, despite minimally invasive colorectal surgery, the incidence of postoperative IH remains high, particularly when the specimen extraction site is located at the midline. According to previous reports, the incidence of IH is approximately 16% [3, 4, 5, 6]. The surgical technique used to close abdominal wall incisions is a notable risk factor for the development of IH. Recent studies have suggested that the use of a continuous small‐bite suturing technique with slowly absorbable sutures for the closure of elective midline incisions can reduce the risk of IH [7, 8, 9, 10].

In this context, the development of barbed suture materials represents an important advancement in wound‐closure surgery and provides a safe and effective alternative to conventional sutures. Their main advantages include a shorter closure time compared to conventional sutures, as they eliminate the need for knot tying and assistance during suturing, and have a more even distribution of tension along the wound [11]. Additionally, compared to conventional slowly absorbable sutures, barbed sutures are associated with a lower risk of evisceration after abdominal fascial closure in emergent surgery [12]. Moreover, recent studies reported that barbed sutures were associated with a reduced incidence of IH compared with interrupted slowly absorbable sutures after minimally invasive colorectal surgery [13, 14]. However, whether barbed sutures are superior to the continuous non‐barbed slowly absorbable sutures recommended in the current guidelines for preventing IH after abdominal surgery remains unclear. Therefore, in this study, we aimed to compare the incidence of IH between using barbed and continuous non‐barbed slowly absorbable monofilament sutures in minimally invasive gastroenterological surgeries.

## 2 Methods

### 2.1 Patient Populations

Patients who underwent elective minimally invasive gastroenterological surgery requiring a midline incision, including the umbilical region for specimen extraction, at our hospital between January 2021 and January 2025 were included in this study. Medical records of all eligible patients were retrospectively reviewed. The exclusion criteria were as follows: Preoperative IH, conversion to open surgery, data regarding the suturing device for closure of the midline incision were unavailable, the specimen extraction site was closed using sutures other than barbed sutures or continuous slowly absorbable monofilament sutures, additional abdominal surgery using the same midline incision within 1 year of the initial surgery, and lack of preoperative or postoperative computed tomography imaging. The following patient data were obtained from medical records: Age, sex, body mass index (BMI), C‐reactive protein levels, prognostic nutritional index (PNI), American Society of Anesthesiologists physical status (ASA‐PS), Eastern Cooperative Oncology Group performance status (ECOG‐PS), comorbidities (diabetes mellitus [DM], chronic obstructive pulmonary disease [COPD], and aortic aneurysms), abdominal surgical history requiring midline incision, rectus diastasis (RD), primary umbilical hernia, medication including corticosteroids or immunosuppressive drugs, and history of perioperative chemotherapy. PNI was calculated using Onodera's formula: PNI = 10 × serum albumin level (g/dL) + 0.005 × total lymphocyte count (/μL) in peripheral blood [15]. Surgical information included the surgical organ system (gastrointestinal surgery or hepato‐biliary‐pancreatic [HBP] surgery), operative time, intraoperative blood loss, type of suture used to close the specimen extraction site, postoperative complication, superficial or deep incisional surgical site infection (SSI), and postoperative hospital stay. Postoperative complications were classified according to the Clavien–Dindo classification [16]. Patients were divided into two groups according to the suture used for fascial closure of the specimen extraction site: The continuous slowly absorbable monofilament suture group (PC group) and the barbed suture group (ST group). The incidence of IH 1 year postoperatively was compared between the two groups. IH was diagnosed using abdominal computed tomography (CT), and the lengths and widths of the IH defects were measured. Abdominal CT imaging performed at 1 year after surgery was used for assessment, allowing a window of ±3 months. Information regarding symptoms attributable to IH and subsequent hernia repair was retrospectively obtained from the medical records throughout the available postoperative follow‐up period.

The study protocol was approved by the institutional review board of Kobe City Medical Center West Hospital (approval no. 24028). Informed consent was obtained from the patients using the opt‐out method owing to the retrospective design of the study, with no risk to the participants.

### 2.2 Surgical Procedures

In the PC group, the rectal fascia was closed using a single continuous triclosan‐coated polydioxanone suture caliber 1 (PDS Plus; Johnson & Johnson). A single anchoring suture was first placed at one end of the incision. Continuous fascial closure was then performed from the opposite end, and the running suture was tied to the anchoring suture to complete the closure.

In the ST group, the rectal fascia was closed using a single continuous suture with a triclosan‐coated barbed suture caliber 1 (STRATAFIX Symmetric PDS Plus; Johnson & Johnson) according to the manufacturer's instructions, without additional PDS sutures.

Although the choice of suture material was left to the discretion of the participating surgeon, the fascial closure technique was generally standardized as described above and gradually changed over the study period as barbed sutures became routinely adopted in our institution. No dissection of the surrounding tissue was routinely performed to expose the fascia before closure.

### 2.3 Definitions

According to the European Hernia Society guidelines, IH was defined as any abdominal wall gap with or without bulging in the area of a postoperative scar perceptible on axial CT images [17]. IH was evaluated by four surgeons (TT, TU, KI, and TK) who were blinded to the methods of abdominal wall closure. The linea alba width was measured on preoperative axial CT images at the point of maximum separation of the two rectus abdominis muscles. RD was defined as separation of the linea alba ≥ 2 cm [18]. The fascial defect at the umbilicus was also assessed on preoperative axial CT images, and primary umbilical hernia was defined as a fascial defect of ≥ 1 cm in width.

### 2.4 Statistical Analysis

Categorical variables are expressed as numbers and percentages, and continuous variables are expressed as medians with ranges. The primary propensity score model included preoperative variables considered clinically relevant to treatment selection or the risk of IH, including age, sex, BMI, smoking status, ASA‐PS, ECOG‐PS, PNI, comorbidities, surgical organ system, history of midline abdominal surgery, presence of RD, presence of primary umbilical hernia, use of steroids or immunosuppressive drugs, and use of preoperative chemotherapy as covariates. Based on the estimated propensity scores, inverse probability of treatment weighting (IPTW) was applied to balance the baseline characteristics between the PC and ST groups, and its effects were assessed using standardized mean differences (SMDs), with an absolute SMD of < 0.1 considered indicative of adequate balance. Postoperative short‐term outcomes were compared between the two groups after IPTW adjustment using weighted univariate analysis. Additionally, the incidence of IH after IPTW was compared between the two groups using logistic regression analysis. Odds ratios (ORs) with 95% confidence intervals (CIs) were calculated. The surgical approach (robot‐assisted surgery vs. laparoscopic surgery) was not included in the primary model because the location and management of the specimen extraction incision were identical between the two approaches, and the increased use of robotic surgery largely coincided with the institutional adoption of barbed suture during the later study period. As a sensitivity analysis, the propensity score was re‐estimated after additionally including the surgical approach, and IPTW‐weighted logistic regression was repeated. For the long‐term analysis of IH, the cumulative incidence function was estimated with IH as the event of interest and death without prior IH as a competing event. The association between suture type and the cumulative incidence of IH was evaluated using an IPTW‐weighted Fine–Gray subdistribution hazards model, and subdistribution hazard ratios (sHRs) with 95% CIs were calculated.

Basic statistical analyses were performed using EZR version 1.52 [19], a graphical user interface for R version 4.02 (The R Foundation for Statistical Computing, Vienna, Austria), which is a modified version of the R Commander, enhanced with statistical functions frequently used in biostatistics. Analyses that were not supported by EZR, including IPTW and balance assessment using SMDs, were performed using R version 4.02 with appropriate packages. A two‐sided *p* < 0.05 was considered statistically significant.

## 3 Result

A flowchart of the study is presented in Figure 1. During the study period, 568 patients underwent elective minimally invasive gastroenterological surgery requiring a midline incision for specimen extraction. Of these, 255 patients were included in the analysis, comprising 142 in the PC group and 113 in the ST group. The baseline characteristics of the study population are presented in Table 1. Most of the patients underwent colorectal surgery, whereas only a small proportion underwent HBP procedures (Table S1). A comparison of the baseline characteristics of the PC and ST groups before and after IPTW adjustment is summarized in Table 2. After IPTW adjustment, the baseline patient characteristics were well balanced between the two groups, with all SMDs < 0.1, except for steroid or immunosuppressive drug use and surgical approach.

**FIGURE 1:**
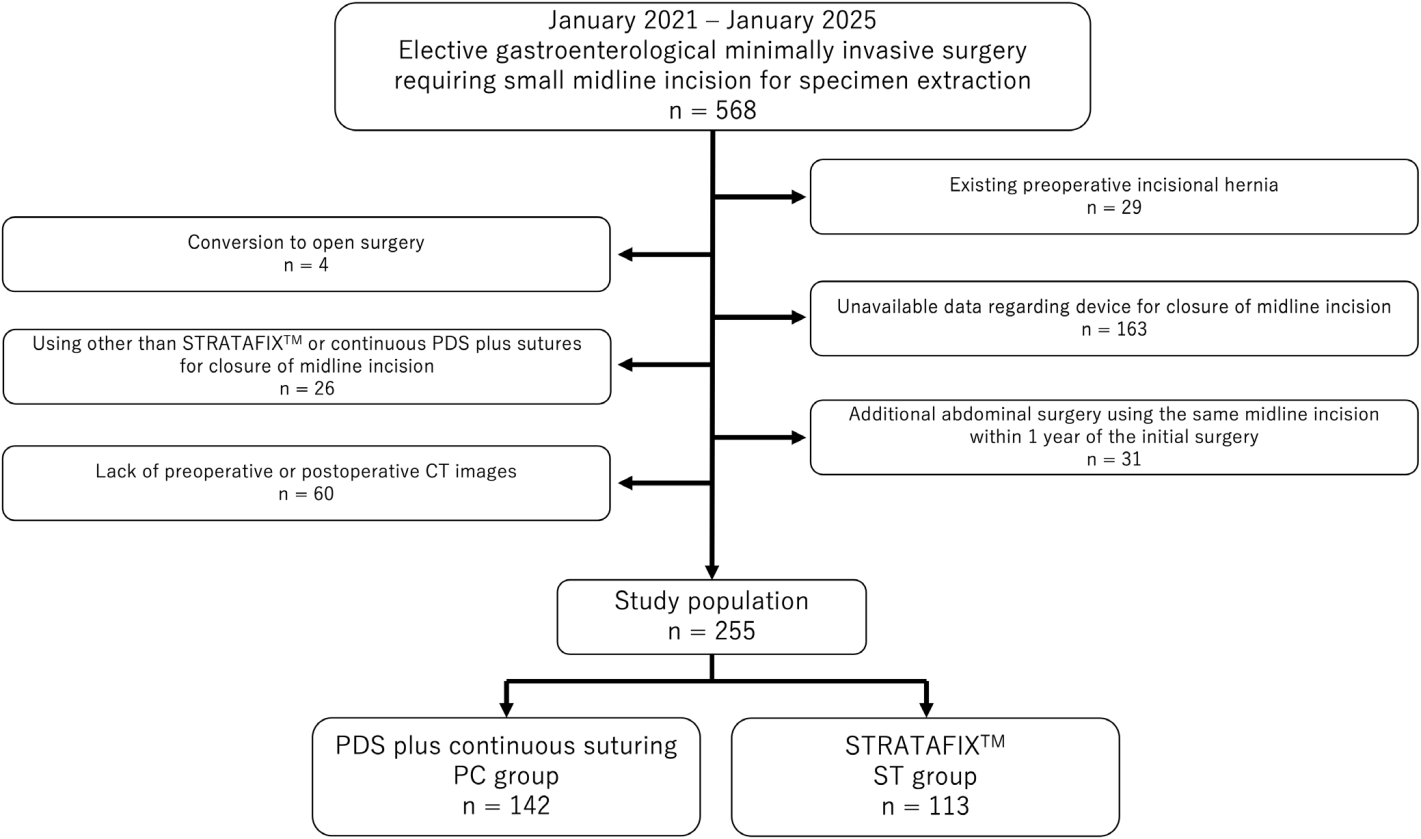
Flowchart of the study.

**TABLE 1:** Baseline characteristics of the study population.

Variable	*n* = 255
Age, years, median (IQR)	75 (68–81)
Sex	
Male	151 (59.2)
Female	104 (40.8)
BMI, kg/m^2^, median (IQR)	22.4 (20.2–24.4)
Active smoking	44 (17.3)
ASA PS ≥ 3	33 (12.9)
ECOG PS ≥ 2	16 (6.3)
Prognostic nutritional index, mean (± SD)	46.5 (± 6.4)
C‐reactive protein, mg/dL, median (IQR)	0.11 (0.10–0.28)
Aortic aneurysm disease	4 (1.6)
Diabetes mellitus	60 (23.5)
Chronic obstructive pulmonary disease	7 (2.7)
History of midline abdominal surgery	66 (25.9)
Rectus diastasis	133 (52.2)
Umbilical hernia	14 (5.5)
Steroid or immunosuppressive drug	2 (0.8)
Preoperative chemotherapy	31 (12.2)
Approach	
Robot‐assisted surgery	81 (31.8)
Laparoscopic surgery	174 (68.2)
Organ system	
Gastrointestinal surgery	245 (96.1)
Hepato‐Biliary‐Pancreatic surgery	10 (3.9)
Suturing device	
Continuous PDS plus suture	142 (55.7)
STRATAFIX	113 (44.3)

*Note:* Values in parentheses are percentages (n [%]).

Abbreviations: ASA PS, American society of anesthesiologists physical status; BMI, body mass index; ECOG PS, eastern cooperative oncology group performance status; IQR, interquartile range; SD, standard deviation.

**TABLE 2:** Baseline characteristics of the PDS plus continuous suturing and STRATAFIX groups.

Variable	Before IPTW			After IPTW		
	PC group *n* = 142	ST group *n* = 113	SMD	PC group *n* = 139.6	ST group *n* = 114.5	SMD
Age ≥ 75	68 (47.9)	60 (53.1)	0.104	69.5 (49.8)	57.1 (49.0)	0.001
Sex			0.133			0.01
Male	80 (56.3)	71 (62.8)		80.5 (57.7)	66.6 (58.2)	
Female	62 (43.7)	42 (37.2)		59.1 (42.3)	47.9 (41.8)	
BMI ≥ 25 kg/m^2^	27 (19.0)	21 (18.6)	0.011	27.4 (19.7)	22.0 (19.3)	0.01
Active smoking	21 (14.8)	23 (20.4)	0.147	23.3 (16.7)	19.0 (16.6)	0.002
ASA PS ≥ 3	22 (15.5)	11 (9.7)	0.174	19.6 (14.1)	15.9 (13.9)	0.005
ECOG PS ≥ 2	4 (2.8)	12 (10.6)	0.316	7.9 (5.7)	7.1 (6.2)	0.022
PNI < 45	56 (39.4)	39 (34.5)	0.102	52.1 (37.3)	44.1 (38.5)	0.025
Aortic aneurysms	2 (1.4)	2 (1.8)	0.029	2.0 (1.4)	1.5 (1.4)	0.006
Diabetes mellitus	36 (25.4)	24 (21.2)	0.097	32.5 (23.3)	27.6 (24.1)	0.02
COPD	5 (3.5)	2 (1.8)	0.109	4.2 (3.0)	4.4 (3.9)	0.047
History of midline abdominal surgery	37 (26.1)	29 (25.7)	0.009	35.7 (25.6)	30.5 (26.7)	0.024
Rectus diastasis	81 (57.0)	52 (46.0)	0.222	74.6 (53.5)	59.3 (51.8)	0.034
Umbilical hernia	9 (6.3)	5 (4.4)	0.085	8.2 (5.9)	7.7 (6.7)	0.033
Steroid or immunosuppressive drug	0 (0.0)	2 (1.8)	0.19	0.0 (0.0)	0.9 (0.8)	0.125
Preoperative chemotherapy	19 (13.4)	12 (10.6)	0.085	17.1 (12.3)	14.5 (12.7)	0.012
Approach			0.641			0.579
Laparoscopic surgery	115 (81.0)	59 (52.2)		112.1 (80.4)	62.1 (54.3)	
Robot‐assisted surgery	27 (19.0)	54 (47.8)		27.4 (19.6)	52.4 (45.7)	
Organ system			0.046			0.005
GI surgery	137 (96.5)	108 (95.6)		134.8 (96.6)	110.7 (96.7)	
HBP surgery	5 (3.5)	5 (4.4)		4.8 (3.4)	3.8 (3.3)	

*Note:* Values in parentheses are percentages (*n* [%]).

Abbreviations: ASA PS, American society of anesthesiologists physical status; BMI, body mass index; COPD, chronic obstructive pulmonary disease; ECOG PS, Eastern Cooperative Oncology Group performance status; GI, gastrointestinal; HBP, hepato‐biliary‐pancreatic; IPTW, inverse probability of treatment weighting; PC group, PDS plus continuous suturing group; PNI, prognostic nutritional index; SMD, standardized mean difference; ST group, STRATAFIX group.

The IPTW‐weighted comparisons of short‐term postoperative outcomes are summarized in Table 3. No significant differences were observed between the two groups in operative time, postoperative complications (Clavien–Dindo≥Grade 2), SSI, and postoperative chemotherapy administration. In the primary IPTW‐weighted analysis, the incidence of IH 1 year after surgery was significantly lower in the ST group than that in the PC group (Table 4). Weighted logistic regression analysis demonstrated that the use of barbed sutures was associated with a reduced risk of IH (OR, 0.46; 95% CI, 0.22–0.99; *p* = 0.047). When the propensity score model was additionally adjusted for the surgical approach, the association remained in favor of barbed sutures but was attenuated and was no longer statistically significant (OR, 0.53; 95% CI, 0.24–1.18; *p* = 0.124).

**TABLE 3:** Short‐term postoperative outcomes of the PDS plus continuous suturing and STRATAFIX groups.

	After IPTW		
	PC group *n* = 139.6	ST group *n* = 114.5	*p*
Operative time, minutes, median (IQR)	313.0 (255.0–393.0)	327.0 (262.0–387.0)	0.257
Blood loss, g, median (IQR)	0.0 (0.0–67.0)	0.0 (0.0–20.0)	0.0073
Any complication (Clavien–Dindo≥Grade 2)	20.8 (14.9)	19.5 (17.0)	0.683
Surgical site infection	8.8 (6.3)	8.9 (7.8)	0.661
Postoperative hospital stay, days, median (IQR)	8.0 (7.0–11.0)	10.0 (8.0–13.0)	< 0.001
Postoperative chemotherapy	36.9 (26.4)	26.9 (23.5)	0.615

*Note:* Values in parentheses are percentages (*n* [%]).

Abbreviations: IPTW, inverse probability of treatment weighting; IQR, interquartile range; PC group, PDS plus continuous suturing group; ST group, STRATAFIX group.

**TABLE 4:** Weighted logistic regression analysis of the incidence of incisional hernia 1 year postoperatively.

	After IPTW			
	PC group *n* = 139.6	ST group *n* = 114.5	Odds ratio (95% CI)	*p*
Incisional hernia	28.7 (20.6)	12.2 (10.7)	0.46 (0.22–0.99)	0.047

*Note:* Values in parentheses are percentages (*n* [%]).

Abbreviations: CI, confidence interval; IPTW, inverse probability of treatment weighting; PC group, PDS plus continuous suturing group; ST group, STRATAFIX group.

Among the 42 patients who developed IH at 1 year postoperatively (31 and 11 in the PC and ST groups, respectively), the length and width of the IH defects were compared between the groups using univariate analysis. The median length (interquartile range [IQR]) was 39.6 mm (33.3–63.4 mm) in the PC group and 39.0 mm (31.5–53.1 mm) in the ST group (*p* = 0.539). The median width (IQR) was 34.7 mm (25.9–45.5 mm) in the PC group and 37.3 mm (23.1–43.5 mm) in the ST group (*p* = 0.597).

Subgroup analyses were performed to evaluate the consistency of treatment effects across clinically relevant patient characteristics (Figure 2). The association between barbed suture closure and a lower incidence of IH was generally consistent across the subgroups defined by age, DM, RD, BMI, prior abdominal surgery, and surgical approach, with no significant interactions observed. Patients with COPD and aortic aneurysms were not included in the subgroup analyses because of the small number of affected patients. The baseline characteristics of patients with and without IH are summarized in Table S2.

**FIGURE 2:**
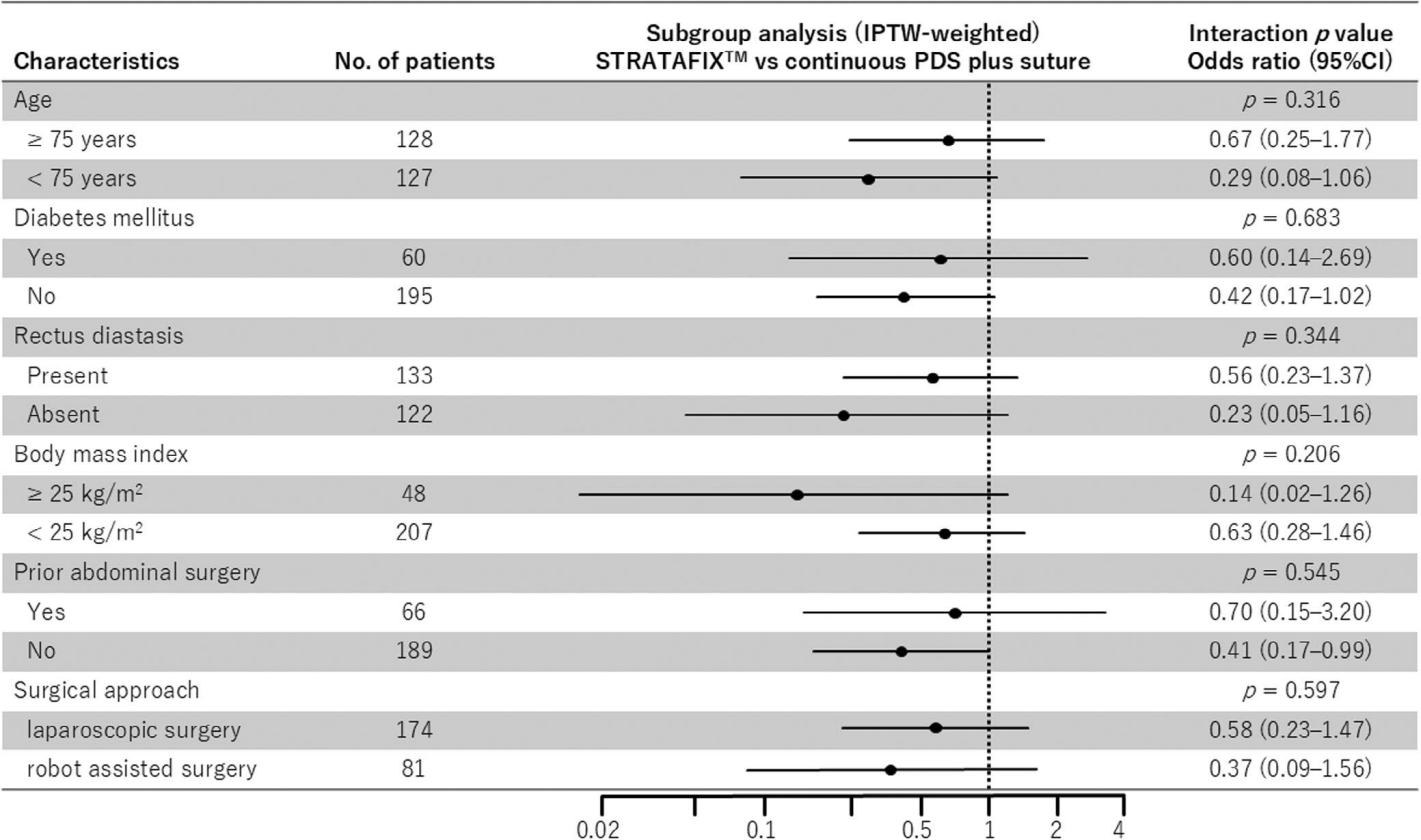
Subgroup analysis of incisional hernia incidence at 1 year after surgery. Forest plot showing the inverse probability of treatment weighting (IPTW)–adjusted odds ratios (ORs) for postoperative incisional hernia comparing the ST group with the PC group across predefined subgroups, including age (≥ 75 vs. < 75 years), diabetes mellitus, rectus diastasis, body mass index (BMI ≥ 25 vs. < 25 kg/m^2^), prior abdominal surgery, and surgical approach. Points indicate IPTW‐weighted ORs, and horizontal lines represent 95% confidence intervals (CIs). ORs < 1.0 favor the ST group.

During the available postoperative follow‐up period, 55 patients developed IH and 20 died without prior IH. Among the patients who developed IH, eight (14.5%) had symptoms such as abdominal distention, while the remaining 47 (85.5%) were asymptomatic. All eight symptomatic patients were in the PC group, of whom seven underwent elective surgical IH repair during the follow‐up period. The median time (range) from index surgery to symptom onset was 15 months (7–29 months), and the median time (range) to hernia repair was 13 months (7–30 months). The median clinical follow‐up duration (range) in the PC group and the ST group was 38.5 months (14–64 months) and 21 months (9–33 months), respectively. In the IPTW‐weighted competing‐risks analysis, with death without prior IH considered a competing event, cumulative incidence of IH was lower in the ST group than in the PC group (sHR, 0.48; 95% CI, 0.25–0.90; *p* = 0.022) (Figure 3). The IPTW‐weighted cumulative incidence of IH at 12, 24, and 36 months was 19.6%, 24.6%, and 26.8%, respectively, in the PC group and 9.0%, 15.7%, and 15.7%, respectively, in the ST group.

**FIGURE 3:**
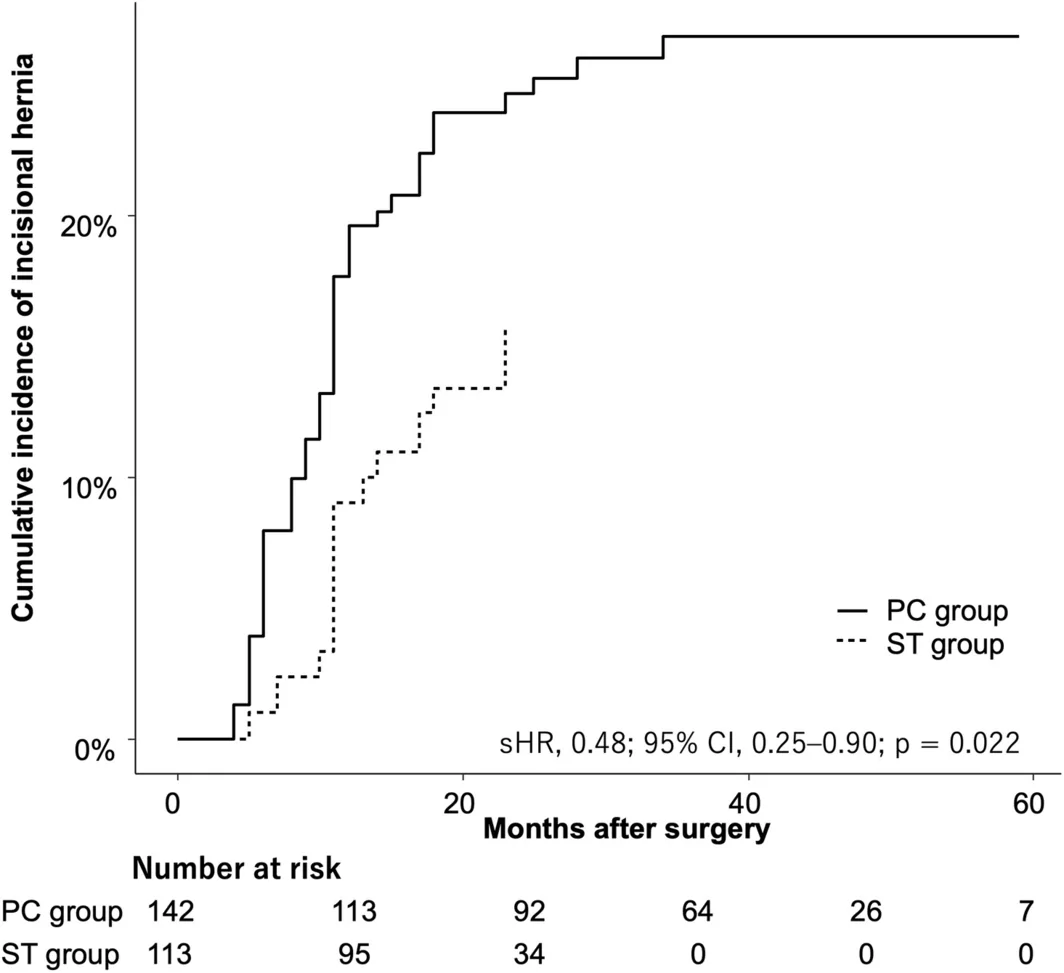
IPTW‐weighted cumulative incidence of incisional hernia. IPTW‐weighted cumulative incidence curves for incisional hernia in the PC and ST groups, with death without prior incisional hernia considered a competing event. The *p*‐value was calculated using an IPTW‐weighted Fine–Gray subdistribution hazards model. Numbers at risk represent the unweighted numbers of patients remaining under observation at each time point. Abbreviations: IPTW, inverse probability of treatment weighting; PC group, PDS plus continuous suturing group; ST group, STRATAFIX group; sHR, subdistribution hazard ratio; CI, confidence interval.

## 4 Discussion

In the present study, we compared the incidence of IH after continuous midline fascial closure using barbed sutures with that using conventional slowly absorbable monofilament sutures in patients undergoing minimally invasive gastroenterological surgery requiring a small midline incision for specimen extraction. After adjustment using IPTW, closure with barbed sutures was associated with a significantly lower incidence of IH at 1 year postoperatively than closure with slowly absorbable monofilament sutures, whereas short‐term postoperative outcomes, including operative time, postoperative complications, and surgical site infection, were comparable between the two groups. Both groups underwent abdominal wall closure using continuous antimicrobial‐coated sutures. Therefore, the presence or absence of barbs may have contributed to the development of IH. Moreover, the lower incidence of IH observed in the ST group may be explained by several characteristics of barbed sutures. First, barbed sutures enable more homogeneous distribution of tension along the suture line, which may reduce segmental ischemia and tissue necrosis, thereby promoting more stable fascial healing [20]. Second, the barbs create multiple fixation points that help maintain secure tissue approximation even under increased intra‐abdominal pressure, preventing loosening of the suture line [12]. Third, barbed sutures have been reported to induce greater fibrous tissue formation around the barbs than conventional non‐barbed sutures, which may further enhance the strength of fascial repair [21]. These properties may have contributed to a reduced risk of developing IH in the ST group.

A major strength of the present study is the use of IPTW based on propensity scores that incorporated known risk factors for IH. Consequently, potential intermediate factors, including SSI and administration of postoperative chemotherapy, were also well balanced between the two groups, enabling a more direct comparison between barbed sutures and slowly absorbable monofilament continuous sutures. Furthermore, the present study incorporated RD, which has increasingly been recognized as a risk factor for IH [22, 23], as a covariate in the propensity score model and adjusted using IPTW. To the best of our knowledge, previous studies comparing barbed sutures with conventional non‐barbed sutures did not account for RD [13, 14]; therefore, by adjusting for this factor, our analysis may have provided a more accurate comparison between the two suturing materials.

In the subgroup analyses, the association between barbed suture closure and a lower incidence of IH was generally consistent across clinically relevant patient subgroups, including age, DM, RD, BMI, prior abdominal surgery, and surgical approach (Figure 2). Although the limited number of events within individual subgroups was likely attributable to the true absence of a treatment effect, some subgroup‐specific estimates did not reach statistical significance. Therefore, even when barbed sutures such as STRATAFIX are used, additional preventive strategies, including the small‐bite technique, maintaining a suture‐to‐wound length ratio of at least 4:1, and avoiding a midline incision for specimen extraction, may be particularly important in patients with risk factors for IH [24]. The small‐bite technique consists of tissue bites of 5–9 mm from the wound edges, incorporating the aponeurosis only, with stitches placed approximately 5 mm apart to ensure adequate distribution of tension [24]. Among the 42 patients who developed IH, eight were symptomatic, all of whom were in the PC group. We hypothesized that the higher proportion of symptomatic cases in the PC group might be related to a larger hernia defect size between the groups. However, no significant differences in either the length or width of the IH defect were observed between the two groups. These findings suggest that the presence of symptoms may not be directly associated with the defect size. Differences in clinical follow‐up duration between the two groups may have influenced the incidence of symptomatic IH and IH repair. Furthermore, as the data were retrospectively extracted from electronic medical records, definitive conclusions could not be drawn.

In this study, the proportion of robot‐assisted surgeries was higher in the ST group than in the PC group. The estimated association was attenuated after the surgical approach was additionally incorporated into the propensity score model, although the direction of the effect remained in favor of barbed sutures. Robotic surgery and barbed suture use both increased substantially during the later study period, resulting in a strong correlation between the surgical approach, closure material, and calendar time. Because the location and closure of the specimen extraction incision were standardized irrespective of the surgical approach, robotic surgery itself may not directly explain the occurrence of IH. Rather, its inclusion may have partly captured secular changes associated with the introduction of barbed sutures and reduced the precision of the effect estimate. Nevertheless, residual confounding by calendar time and other concurrent changes in surgical practice cannot be excluded. Previous studies have demonstrated no significant difference in the incidence of IH between the robotic and laparoscopic approaches [13]. Steroid or immunosuppressive drug use, which are known risk factors for IH, was more frequent in the ST group than in the PC group. Although the incidence of IH was lower in the ST group than in the PC group, suggesting that the observed benefit of barbed sutures is unlikely to be explained by residual confounding factors.

Despite the positive outcomes, this study has some limitations. First, this study was a retrospective study conducted at a single center; therefore, selection bias may have occurred, limiting the generalizability of the findings. Although this study included patients undergoing various minimally invasive gastroenterological procedures, only a small number underwent HBP surgery. Therefore, the generalizability of our findings to patients undergoing minimally invasive HBP surgery may be limited. Second, although the fascial closure technique was generally standardized at our institution, detailed surgeon‐specific technical variables, including the suture‐to‐wound length ratio, bite width, number of stitches, and individual surgeon experience, were not available because of the retrospective design. Therefore, the potential influence of surgeon‐related factors and unmeasured technical differences on the development of IH could not be completely excluded; however, the participating surgeons generally performed fascial closure with the recommended target values in mind. Third, long‐term follow‐up was not performed in this study. Fink et al. reported a significant increase in the incidence of IH between one and 3 years after surgery [25], indicating that the incidence of IH may have been underestimated in our study. They also suggested that a minimum follow‐up period of 3 years was required for studies evaluating postoperative IH after midline incision. Furthermore, although the primary endpoint was assessed uniformly using CT approximately 1 year after surgery, IH may develop beyond the first postoperative year. To further evaluate the longer‐term incidence of IH, we performed an additional IPTW‐weighted competing‐risks analysis using all available follow‐up data, with death without prior IH considered a competing event. The cumulative incidence of IH remained lower in the ST group than in the PC group, and the effect estimate was consistent with that of the primary analysis. However, these findings should be interpreted cautiously. The duration of clinical follow‐up differed between the groups because STRATAFIX was introduced later in the study period. Therefore, the primary findings of the present study principally reflect the incidence of CT‐detected IH within the first postoperative year, and the additional competing‐risks analysis should not be interpreted as demonstrating a definitive long‐term preventive effect of barbed sutures. Finally, IH was diagnosed based on the CT findings. Although CT allows the objective detection of small or subclinical hernias, this approach may also identify asymptomatic hernias, and the clinical relevance of CT‐detected IH remains controversial.

Nevertheless, the present study provides clinically relevant information on the potential role of barbed sutures in reducing IH after minimally invasive gastroenterological surgery. Further investigations are warranted to validate these results and confirm their generalizability.

## 5 Conclusion

In this study, after adjustment using IPTW, closure with barbed sutures was associated with a significantly lower incidence of IH at 1 year postoperatively than closure with slowly absorbable monofilament sutures in patients undergoing minimally invasive gastroenterological surgery requiring a small midline incision for specimen extraction. This finding suggests that it is important to use barbed sutures such as STRATAFIX sutures, identify patient‐related risk factors for IH, and perform abdominal wall closure using appropriate suturing techniques to prevent IH.

## Author Contributions

**Shusaku Honma:** conceptualization, writing – review and editing, writing – original draft, formal analysis, data curation, methodology. **Takashi Takashima:** data curation, investigation, writing – review and editing. **Tatsuhi Ushikubo:** data curation, investigation, writing – review and editing. **Kana Ishikawa:** data curation, investigation, writing – review and editing. **Takashi Kumode:** data curation, investigation, writing – review and editing. **Takahisa Suzuki:** data curation, writing – review and editing. **Teppei Murakami:** data curation, writing – review and editing. **Shinichi Hosokawa:** data curation, writing – review and editing. **Sanae Nakajima:** writing – review and editing, supervision.

## Funding

The authors have nothing to report.

## Ethics Statement

The study protocol was approved by the Institutional Review Board of Kobe City Medical Center West Hospital (approval no. 24028) and performed in accordance with the Declaration of Helsinki.

## Consent

The requirement for informed consent was waived by the Institutional Review Board of Kobe City Medical Center West Hospital owing to the retrospective nature of the study.

## Conflicts of Interest

The authors declare no conflicts of interest.

## Supporting information


**Table S1:** Detailed classification of operative procedures.

**Table S2:** Comparison of patients with and without incisional hernia.

## Data Availability

Data sharing not applicable to this article as no datasets were generated or analysed during the current study.

## References

[1] B. K. Poulose , J. Shelton , S. Phillips , et al., “Epidemiology and Cost of Ventral Hernia Repair: Making the Case for Hernia Research,” Hernia 16, no. 2 (2012): 179–183. 10.1007/s10029-011-0879-9 21904861

[2] J. B. Kössler‐Ebs , K. Grummich , K. Jensen , et al., “Incisional Hernia Rates After Laparoscopic or Open Abdominal Surgery—A Systematic Review and Meta‐Analysis,” World Journal of Surgery 40, no. 10 (2016): 2319–2330. 10.1007/s00268-016-3520-3 27146053

[3] J. Petersson , T. W. Koedam , H. J. Bonjer , et al., “Bowel Obstruction and Ventral Hernia After Laparoscopic Versus Open Surgery for Rectal Cancer in A Randomized Trial (COLOR II),” Annals of Surgery 269, no. 1 (2019): 53–57. 10.1097/SLA.0000000000002790 29746337

[4] G. W. Taylor , D. G. Jayne , S. R. Brown , et al., “Adhesions and Incisional Hernias Following Laparoscopic Versus Open Surgery for Colorectal Cancer in the CLASICC Trial,” British Journal of Surgery 97, no. 1 (2010): 70–78. 10.1002/bjs.6742 20013936

[5] N. Pecorelli , S. Amodeo , M. Frasson , A. Vignali , W. Zuliani , and M. Braga , “Ten‐Year Outcomes Following Laparoscopic Colorectal Resection: Results of a Randomized Controlled Trial,” International Journal of Colorectal Disease 31, no. 7 (2016): 1283–1290. 10.1007/s00384-016-2587-5 27090804

[6] F. P. J. den Hartog , S. van Egmond , M. M. Poelman , et al., “The Incidence of Extraction Site Incisional Hernia After Minimally Invasive Colorectal Surgery: A Systematic Review and Meta‐Analysis,” Colorectal Disease 25, no. 4 (2023): 586–599. 10.1111/codi.16455 36545836

[7] S. Morarasu , S. Lunca , L. O'Brien , et al., “Small‐Bites Versus Large‐Bites Closure of Midline Laparotomies: A Systematic Review and Meta‐Analysis,” Colorectal Disease 27, no. 3 (2025): e70073. 10.1111/codi.70073 PMC11933735 40129086

[8] R. van den Berg , L. Visscher , A. G. Menon , E. B. Deerenberg , and P. J. Tanis , “Suture Techniques and Materials for Fascial Closure of Abdominal Wall Incisions: A Comprehensive Meta‐Analysis,” Ann Surg Open 6, no. 1 (2025): e548. 10.1097/AS9.0000000000000548 PMC11932593 40134474

[9] A. Alrashidi , A. W. Hageen , M. Rifai , et al., “Small Bites Versus Large Bites for Closure of Midline Abdominal Incisions: A Systematic Review and Meta‐Analysis With Trial Sequential Analysis of Randomized Controlled Trials,” World Journal of Surgery 49, no. 6 (2025): 1449–1465. 10.1002/wjs.12619 40354104

[10] F. E. Muysoms , S. A. Antoniou , K. Bury , et al., “European Hernia Society Guidelines on the Closure of Abdominal Wall Incisions,” Hernia 19, no. 1 (2015): 1–24. 10.1007/s10029-014-1342-5 25618025

[11] F. Berrevoet , L. van Cauteren , N. Gunja , et al., “Clinical Outcomes of Triclosan‐Coated Barbed Suture in Open Hernia Repair: A Retrospective Cohort Study,” Hernia 28, no. 4 (2024): 1239–1247. 10.1007/s10029-024-03012-0 PMC11297053 38609582

[12] J. Ruiz‐Tovar , C. Llavero , M. Jimenez‐Fuertes , M. Duran , M. Perez‐Lopez , and A. Garcia‐Marin , “Incisional Surgical Site Infection After Abdominal Fascial Closure With Triclosan‐Coated Barbed Suture vs Triclosan‐Coated Polydioxanone Loop Suture vs Polydioxanone Loop Suture in Emergent Abdominal Surgery: A Randomized Clinical Trial,” Journal of the American College of Surgeons 230, no. 5 (2020): 766–774. 10.1016/j.jamcollsurg.2020.02.031 32113031

[13] K. Sato , T. Mukai , Y. Kamei , et al., “Continuous Fascial Closure Using Absorbable Barbed Sutures Lowers the Risk of Incisional Hernia After Minimally Invasive Colorectal Surgery: A Consecutive Single‐Surgeon Retrospective Cohort Study,” Surgical Endoscopy 40 (2026): 3003–3011. 10.1007/s00464-026-12577-z 41559336

[14] K. Nagayoshi , Y. Mizuuchi , T. Fujimoto , et al., “Incisional Hernia After Continuous Barbed Versus Interrupted Non‐Barbed Sutures for Midline Fascial Closure in Minimally Invasive Colorectal Cancer Surgery: A Propensity Score‐Matched Analysis,” Hernia 30, no. 1 (2026): 78. 10.1007/s10029-025-03582-7 41627512

[15] T. Onodera , N. Goseki , and G. Kosaki , “Prognostic Nuturition Index in Gastrointestinal Surgery of Malnourished Cancer Patients,” Nihon Geka Gakkai Zasshi 85, no. 9 (1984): 1001–1005. 6438478

[16] D. Dindo , N. Demartines , and P. A. Clavien , “Classification of Surgical Complications: A New Proposal With Evaluation in a Cohort of 6336 Patients and Results of a Survey,” Annals of Surgery 240, no. 2 (2004): 205–213. 10.1097/01.sla.0000133083.54934.ae PMC1360123 15273542

[17] F. E. Muysoms , M. Miserez , F. Berrevoet , et al., “Classification of Primary and Incisional Abdominal Wall Hernias,” Hernia 13, no. 4 (2009): 407–414. 10.1007/s10029-009-0518-x PMC2719726 19495920

[18] P. Hernández‐Granados , N. A. Henriksen , F. Berrevoet , et al., “European Hernia Society Guidelines on Management of Rectus Diastasis,” British Journal of Surgery 108, no. 10 (2021): 1189–1191. 10.1093/bjs/znab128 PMC10364860 34595502

[19] Y. Kanda , “Investigation of the Freely Available Easy‐To‐Use Software “EZR” for Medical Statistics,” Bone Marrow Transplantation 48, no. 3 (2013): 452–458. 10.1038/bmt.2012.244 PMC3590441 23208313

[20] V. Pla‐Martí , J. Martín‐Arévalo , D. Moro‐Valdezate , et al., “Triclosan‐Coated Barbed Sutures in Elective Laparoscopic Colorectal Cancer Surgery: A Propensity Score Matched Cohort Study,” Surgical Endoscopy 37, no. 1 (2023): 209–218. 10.1007/s00464-022-09418-0 PMC9839817 35918550

[21] M. T. Villa , L. E. White , M. Alam , S. S. Yoo , and R. L. Walton , “Barbed Sutures: A Review of the Literature,” Plastic and Reconstructive Surgery 121, no. 3 (2008): 102e–108e. 10.1097/01.prs.0000299452.24743.65 18317092

[22] A. T. Booth , K. J. Gillen , I. Visintin , et al., “Diastasis Recti Is Associated With Incisional Hernia After Midline Abdominal Surgery,” Hernia 27, no. 2 (2023): 363–371. 10.1007/s10029-022-02676-w 36136228

[23] S. Honma , K. Ishikawa , T. Kumode , et al., “Rectus Diastasis Is a Risk Factor for Incisional Hernia After Robot Assisted Laparoscopic Radical Prostatectomy for Prostate Cancer,” Hernia 29, no. 1 (2025): 233. 10.1007/s10029-025-03419-3 40668292

[24] E. B. Deerenberg , N. A. Henriksen , G. A. Antoniou , et al., “Updated Guideline for Closure of Abdominal Wall Incisions From the European and American Hernia Societies,” British Journal of Surgery 109, no. 12 (2022): 1239–1250. 10.1093/bjs/znac302 PMC10364727 36026550

[25] C. Fink , P. Baumann , M. N. Wente , et al., “Incisional Hernia Rate 3 Years After Midline Laparotomy,” British Journal of Surgery 101, no. 2 (2014): 51–54. 10.1002/bjs.9364 24281948

