# Supplementary material for: Barbed Sutures Are Associated With a Lower Incidence of Incisional Hernia Compared With Continuous Non‐Barbed Monofilament Sutures After Minimally Invasive Gastroenterological Surgery
Source: Ann Gastroenterol Surg. 2026 Oct 1:10.1002/ags3.70294. Online ahead of print. doi: 10.1002/ags3.70294 (PMC13631104; doi:10.1002/ags3.70294)
Supplement: Supplementary file 1 [file AGS3-9999-0-s001.docx]

Supplementary Table 1 Detailed classification of operative procedures

|  | PC group  n = 142 | ST group  n = 113 | Total  n = 255 |
| --- | --- | --- | --- |
| Gastrectomy | 30 (21.1) | 24 (21.2) | 54 (21.2) |
| Small bowel resection | 1 (0.7) | 2 (1.8) | 3 (1.2) |
| Right colectomy | 39 (27.5) | 26 (23.0) | 65 (25.5) |
| Left colectomy | 29 (20.4) | 17 (15.0) | 46 (18.0) |
| Rectal resection | 38 (26.8) | 39 (34.5) | 77 (30.2) |
| Hepatectomy | 2 (1.4) | 5 (4.4) | 7 (2.7) |
| Pancreatectomy | 3 (2.1) | 0 (0) | 3 (1.2) |

Abbreviations: PC group, PDS plus continuous suturing group; ST group, STRATAFIX^TM^ group;

Values in parentheses are percentages (n [%]).
