# Supplementary material for: Barbed Sutures Are Associated With a Lower Incidence of Incisional Hernia Compared With Continuous Non‐Barbed Monofilament Sutures After Minimally Invasive Gastroenterological Surgery
Source: Ann Gastroenterol Surg. 2026 Oct 1:10.1002/ags3.70294. Online ahead of print. doi: 10.1002/ags3.70294 (PMC13631104; doi:10.1002/ags3.70294)
Supplement: Supplementary file 2 [file AGS3-9999-0-s002.docx]

Supplementary Table 2 Comparison of patients with and without incisional hernia

| Variable | Non-IH  n = 213 | IH  n = 42 | *p* value |
| --- | --- | --- | --- |
| Age > 75 | 106 (49.8) | 22 (52.4) | 0.866 |
| Sex  Male  Female | 130 (61.0)  83 (39.0) | 21 (50)  21 (50) | 0.229 |
| BMI > 25 kg/m^2^ | 37 (17.4) | 11 (26.2) | 0.197 |
| Active smoking | 38 (17.8) | 6 (14.3) | 0.661 |
| ASA PS ≥ 3 | 29 (13.6) | 4 (9.5) | 0.618 |
| ECOG PS ≥ 2 | 15 (7.0) | 1 (2.4) | 0.483 |
| PNI < 45 | 79 (37.1) | 16 (38.1) | 1 |
| CRP, mg/dl, median (IQR) | 0.11 (0.10–0.27) | 0.12 (0.10–0.36) | 0.563 |
| Aortic aneurysm disease | 3 (1.4) | 1 (2.4) | 0.515 |
| Diabetes mellitus | 50 (23.5) | 10 (23.8) | 1 |
| Chronic obstructive pulmonary disease | 4 (1.9) | 3 (7.1) | 0.09 |
| History of midline abdominal surgery | 57 (26.8) | 9 (21.4) | 0.565 |
| Rectus diastasis | 101 (47.4) | 32 (76.2) | 0.001 |
| Umbilical hernia | 8 (3.8) | 6 (14.3) | 0.015 |
| Steroid or immunosuppressive drug | 2 (0.9) | 0 (0) | 1 |
| Preoperative chemotherapy | 28 (13.1) | 3 (7.1) | 0.437 |
| Approach  Robot assisted surgery  Laparoscopic surgery | 72 (33.8)  141 (66.2) | 9 (21.4)  33 (78.6) | 0.147 |
| Organ system  GI surgery  HBP surgery | 203 (95.3)  10 (4.7) | 42 (100)  0 (0) | 0.376 |
| Suturing device  Continuous PDS plus suture  STRATAFIX^TM^ | 111 (52.1)  102 (47.9) | 31 (73.8)  11 (26.2) | 0.011 |
| Operative time, minutes, median (IQR) | 318.0 (256.0–390.0) | 318.5 (257.3–408.0) | 0.701 |
| Blood loss, g, median (IQR) | 0.0 (0.0–50.0) | 0.0 (0.0–48.8) | 0.64 |
| Any complication (CD ≥ grade 2) | 30 (14.1) | 8 (19.0) | 0.476 |
| Surgical site infection | 10 (4.7) | 7 (16.7) | 0.011 |
| POHS, days, median (IQR) | 9 (7–11) | 8 (7–12) | 0.691 |
| Postoperative chemotherapy | 48 (22.5) | 15 (35.7) | 0.08 |
| Follow up duration, months median (IQR) | 28.0 (20.0–39.0) | 33.5 (23.3–41.8) | 0.084 |

Abbreviations: IH, incisional hernia; BMI, body mass index; ASA PS, American Society of Anesthesiologists physical status; ECOG PS, Eastern Cooperative Oncology Group performance status; PNI, prognostic nutritional index; CRP, C-reactive protein; GI, gastrointestinal; HBP, hepato-biliary-pancreatic; CD, Clavien–Dindo; POHS, post-operative hospital stay; IQR, inter-quartile range

Values in parentheses are percentages, n (%).
